# Comparing surgical interventions for intertrochanteric hip fracture by blood loss and operation time: a network meta-analysis

**DOI:** 10.1186/s13018-018-0852-8

**Published:** 2018-06-22

**Authors:** Zhengan Hao, Xifeng Wang, Xingqun Zhang

**Affiliations:** 1 Department of Orthopaedics, Yuhang Branch of the Second Affiliated Hospital of Zhejiang University, Hangzhou, China; 2 Department of Respiration, Yuhang Branch of the Second Affiliated Hospital of Zhejiang University, Hangzhou, China

**Keywords:** Intertrochanteric hip fracture, Surgical intervention, Meta-analysis

## Abstract

**Background:**

Multiple operative treatments are available for the fixation of intertrochanteric femoral fractures. This analysis was conducted to provide guidance on the appropriate clinical choice to accommodate individual patients.

**Methods:**

A systematic review was performed to identify relevant articles in databases. Randomized controlled trials (RCTs) of adults with intertrochanteric femoral fractures were eligible if they compared 2 or more of the following interventions: proximal femoral nail anti-rotation (PFNA), percutaneous compression plate (PCCP) use, dynamic hip screw (DHS) fixation, gamma nail (GN) fixation, and artificial femoral head replacement (FHR). Bayesian network meta-analysis was performed to simultaneously compare all treatment methods.

**Results:**

In total, 24 active-comparator studies involving 3097 participants were identified. Across all populations, greater reductions in blood loss and operation time were observed for PFNA than for other treatments. In terms of bleeding, more blood loss was observed for DHS use than for the PFNA (SMD, 1.96; 95% CI, 1.01–1.96), PCCP (SMD, 1.26; 95% CI, 0.31–2.20), and GN (SMD, 0.26; 95% CI, − 0.35–0.87) techniques. However, a more beneficial effect was observed for DHS use than for FHR (SMD, − 0.23; 95% CI, − 1.26–0.81). DHS use resulted in a significantly longer duration of operation time than the PFNA (SMD, 0.75; 95% CI, − 0.02–0.75), PCCP (SMD, 0.61; 95% CI, − 0.20–1.44), and GN (SMD, 0.25; 95% CI, − 0.26–0.77) techniques. Similarly, greater reductions in operation time were observed for DHS use than for FHR (SMD, − 0.12; 95% CI, − 1.15–0.91).

**Conclusions:**

The findings provide supporting evidence demonstrating the superiority of PFNA over other treatments for intertrochanteric femoral fracture. PFNA treatment results in the lowest amount of blood loss and the shortest operation time. These findings add to the existing knowledge of intertrochanteric femoral fracture treatment options.

## Background

The incidence of intertrochanteric hip fracture, which accounts for 31–51% of proximal femoral fractures [1], has increased during the last decade. Accompanied by physical deterioration, this type of fracture is prevalent among geriatric patients. Furthermore, most patients with this type of fracture experience medical comorbidities, which can worsen complications if left untreated. Consequently, early surgery is considered the best option for these patients by the majority of clinicians worldwide. The advantages of surgery include the restoration of anatomical alignment and early rehabilitation [2]. Moreover, early surgery can reduce the risk of complications.

Fixation with dynamic hip screws (DHS) is generally considered the standard management option for treating these fractures and has gained extensive acceptance [3, 4]. However, various types of implants are available, including extramedullary and intramedullary devices. Consequently, the preferred treatment option remains controversial due to the lack of randomized controlled trials (RCTs) comparing these therapies. Accordingly, an analysis that integrates evidence of current on treatment options for intertrochanteric hip fracture is needed.

A traditional pairwise meta-analysis cannot simultaneously include various types of prevalent therapies. To identify the optimal treatment method, we compared all candidate therapies in the same analysis by performing a network of treatments meta-analysis involving a direct analysis and a combined analysis. We aimed to comprehensively integrate the current evidence regarding blood loss and operation time outcomes from operative interventions in patients with intertrochanteric fractures.

## Materials and methods

### Search strategy

EMBASE, MEDLINE, CNKI, and the Cochrane Library were searched. All searches were limited to RTCs involving humans. Searches were not limited by language, publication date, or publication status. We also reviewed the reference lists of all selected studies to identify any additional relevant articles. The searches incorporated the following keywords: “intertrochanteric fractures,” “proximal femoral nail anti-rotation,” “dynamic hip screw,” “gamma nail,” “percutaneous compression plate,” and “artificial femoral head replacement.”

### Study selection

Studies that satisfied the following inclusion criteria were eligible for the analysis: (1) RCTs involving human participants treated for intertrochanteric hip fractures; (2) evaluations including either surgical time or blood loss, with complete data; (3) patients aged over 18 years; (4) the highest-quality study or the most recent publication in cases where data were duplicated; (5) comparison of at least two of the five treatments studied (DHS, PFNA, GN, PCCP, FHR).

### Data extraction and quality assessment

The data were reviewed independently by two extractors. Extractors resolved any disagreements by consensus. The following details were extracted from each study: name of the first author, country of origin, sample size, year of publication, treatments compared, design, population characteristics, outcome measures, and fracture classification. The outcome measures of interest were surgical time and blood loss, as measured by change from baseline. The reviewers evaluated the methodological quality of all individual studies using the JADAD scale [5]. Particularly, the reviewers assessed the procedures of randomization sequence generation, appropriate blinding, allocation concealment, and reporting of withdrawal.

### Statistical analysis

Eligible trials comprising direct and indirect comparisons were summarized qualitatively. In addition, essential clinical outcomes and methodological variables were recorded. We performed a traditional “head-to-head” meta-analysis to calculate the pooled estimates of the standard mean difference (SMD) and 95% confidence intervals (CIs). We conducted the statistical analysis using Stata version 11.0 (Stata Corporation, College Station, TX, USA) and developed a DerSimonian-Laird random effects model for comparisons between two strategies [6]. The Cochrane Q test and the *I*^2^ statistic were assessed to determine the effect of between-study heterogeneity [7, 8]. Begg’s test was also conducted to investigate publication bias [9].

We also performed a random effects Bayesian network meta-analysis to enhance the head-to-head meta-analysis. Using this method, all five treatments could be simultaneously considered. In addition, this method increases statistical power by merging both direct and indirect comparison evidence across all five interventions. Therefore, conclusions could be educed on the relative effectiveness of interventions, such as GN fixation and PCCP use, which have never before been directly compared.

The network meta-analyses were undertaken using Bayesian Markov chain Monte Carlo (MCMC) simulation methods with minimally informative prior distributions. Thousands of simulated iterations were run based on the data and description of the proposed distributions of relevant parameters in WinBUGS software (version 1.4.3. MRC Biostatistics Unite, Cambridge, UK) [10]. The first 10,000 iterations (burn-in) were discarded, and 40,000 further iterations were run. Pooled effect sizes are reported as posterior medians of SMDs, and the corresponding 95% credible intervals (CrIs) were applied using the 2.5th and 97.5th percentiles of the posterior distribution, which can be interpreted similar to conventional 95% CIs [11]. We also calculated the percentage contribution of each effect size in the evidence base, i.e., the probability that each treatment was the most effective therapy or the second most effective therapy, etc. The distribution of ranking probabilities is presented graphically.

Meta-regression analyses were conducted to evaluate whether the publication year and type of fractures could explain the latent heterogeneity. The inconsistency between direct and indirect comparisons was evaluated using the loop-specific method [12]. This approach evaluates the difference (inconsistency factor) and 95% CI, between direct and indirect estimations for a specific comparison. Inconsistency was defined as disagreement between direct and indirect evidence with a 95% CI excluding 0.

## Results

### Eligible studies and characteristics

**Fig. 1:**
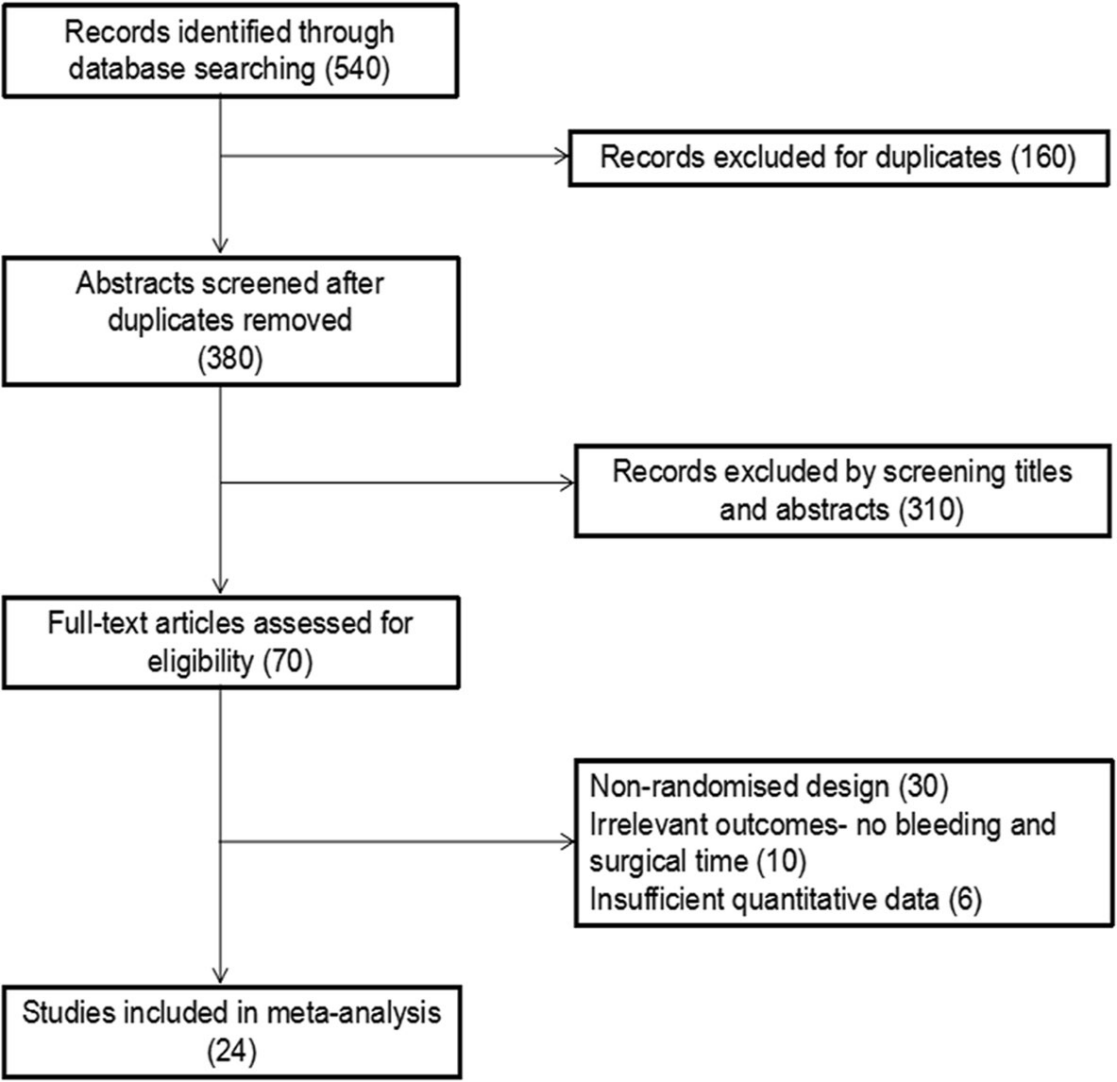
Flowchart of the study selection process

**Fig. 2:**
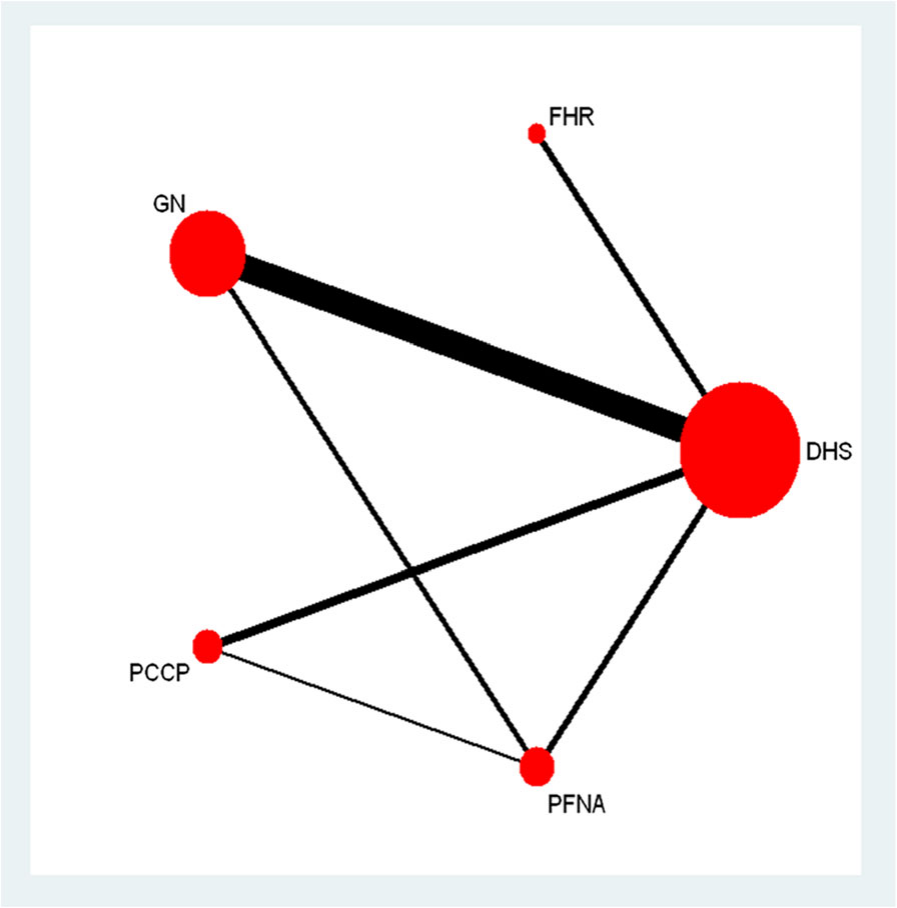
The eligible clinical trials included in the network meta-analysis. The width of the connecting lines is proportional to the number of available head-to-head (direct) comparisons. The size of each node is proportional to the number of randomly assigned participants (sample size)

After reviewing all selected studies, 24 trials involving five treatments met the selection criteria [13–36]. A total of 3097 individuals with sufficient data were included in the quantitative synthesis (meta-analysis). The study selection process, including reasons for exclusion, is presented by a flowchart (Fig. 1). The characteristics of all involved studies are summarized in Table 1. Figure 2 shows all comparisons analyzed within the network. The study sample size varied from 60 to 426 participants, with a median sample size of 129. The mean age of patients varied from 62.2 to 83.6 years. According to the Evans or AO/OTA classification, all fractures were categorized as stable (Evans type I/II and AO/ATO type 31-A1) or unstable (Evans type III–V and AO/ATO type 31-A2/A3). In 7 trials, all selected patients had unstable fractures; in 15 trials, all selected patients had compound fractures, either stable or unstable. We conducted a quality assessment of all included studies (Table 2) and found that the studies had a broad range of overall reporting quality. The proportions of studies with definite descriptions of randomization, allocation concealment, blinding, dropout, and intention-to-treat analysis were 83.33% (20/24), 70.83% (17/24), 62.5% (15/24), 87.5% (21/24), and 91.67% (22/24), respectively. According to meta-regression analyses, blood loss and operation time were not affected by differences across trials in terms of the publication year or type of fractures (Table 3). No evidence of inconsistency between the direct and indirect estimates was observed in this meta-analysis (Table 4). No publication bias was observed among the included studies (Table 5).

**Table 1:** Baseline characteristics of included studies

Author (year)	Country	Intervention	Mean age (years)	Patients (number)	Type of fractures (unstable/stable)
Tang 2009	China	DHS, FHR	80.7	109	109/0
Verettas 2010	Greece	DHS, GN	80.1	118	118/0
Zou 2009	China	DHS, PFNA	65	121	94/27
Xu 2010	China	DHS, PFNA	78.2	106	106/0
Wang 2010	China	DHS, FHR	83.5	60	60/0
Peyser 2007	Israel	DHS, PCCP	80.8	103	60/22
Ahrengart 2002	Sweden	DHS, GN	80	426	222/204
Xu 2010	China	PFNA, GN	76.7	107	40/67
Butt 1995	UK	DHS, GN	78.5	95	30/30
Leung 1992	China	DHS, GN	79.6	186	50/136
Kosygan 2002	England	DHS, PCCP	82.8	108	49/59
Peter 1995	Canada	DHS, GN	80.1	102	58/44
Kukla 1997	Austria	DHS, GN	83.5	120	54/66
Yang 2011	America	DHS, PCCP	76.5	66	NA
Hoffman 1996	New Zealand	DHS, GN	80.9	67	22/45
Garg 2011	India	DHS, PFNA	62.2	81	81/0
Guo 2013	China	PFNA, PCCP	72.9	90	49/41
Brandt 2002	Netherlands	DHS, PCCP	80.9	71	41/30
Aktselis 2014	Greece	DHS, GN	83	71	71/0
Vaquero 2012	Spain	PFNA, GN	83.6	61	61/0
Utrilla 2005	Spain	DHS, GN	80.2	210	54/156
Goldhagen 1994	America	DHS, GN	78	63	NA
Christopher 2001	England	DHS, GN	81	400	193/207
Huang 2006	China	DHS, FHR	72.8	156	43/113

**Table 2:** Methodological quality assessment by adjusted Jadad scale

Author	Random sequence generation	Allocation concealment	Blinding of participants	Dropout Addressed	ITT
Tang 2009	Unclear	Unclear	Unclear	Adequate	Y
Verettas 2010	Adequate	Unclear	Unclear	Adequate	Y
Zou 2009	Inadequate	Unclear	Unclear	Unclear	Y
Xu 2010	Adequate	Adequate	Adequate	Adequate	Y
Wang 2010	Inadequate	Unclear	Unclear	Unclear	N
Peyser 2007	Adequate	Adequate	Adequate	Adequate	Y
Ahrengart 2002	Adequate	Adequate	Adequate	Adequate	Y
Xu 2010	Adequate	Adequate	Adequate	Adequate	Y
Butt 1995	Adequate	Adequate	Adequate	Unclear	Y
Leung 1992	Adequate	Adequate	Inadequate	Adequate	Y
Kosygan 2002	Adequate	Adequate	Adequate	Adequate	Y
Peter 1995	Adequate	Adequate	Adequate	Adequate	Y
Kukla 1997	Adequate	Adequate	Adequate	Adequate	Y
Yang 2011	Adequate	Adequate	Adequate	Adequate	Y
Hoffman 1996	Adequate	Adequate	Adequate	Adequate	Y
Garg 2011	Adequate	Unclear	Unclear	Adequate	N
Guo 2013	Adequate	Adequate	Unclear	Adequate	Y
Brandt 2002	Adequate	Adequate	Adequate	Adequate	Y
Aktselis 2014	Inadequate	Unclear	Unclear	Adequate	Y
Vaquero2012	Adequate	Adequate	Adequate	Adequate	Y
Utrilla 2005	Adequate	Adequate	Adequate	Adequate	Y
Goldhagen 1994	Adequate	Unclear	Unclear	Unclear	Y
Christopher	Adequate	Adequate	Adequate	Adequate	Y
Huang 2006	Adequate	Adequate	Adequate	Adequate	Y

*ITT* intention-to-treat analysis

**Table 3:** Results of the meta-regression

Outcome	Regression coefficient(95%CI)	
	Publication year	Type of fractures
Blood loss	− 0.02 (− 0.11, 0.08)	− 0.70 (− 2.04, 0.64)
Operation time	− 0.32 (− 0.10, 0.04)	− 0.22 (− 1.28, 0.83)

**Table 4:** Assessment of inconsistency between direct and indirect evidence

Loop	Blood loss	Operation time
	Inconsistency 95% CI	Inconsistency 95% CI
DHS,PCCP, PFNA	3.59 (0.00, 7.33)	1.82 (0.00, 5.77)
DHS, GN, PFNA	2.66 (0.00, 4.77)	0.71 (0.00, 2.71)

**Table 5:** Assessment of heterogeneity and publication bias for trials included indirect meta-analyses

Comparisons	Number	Q-test heterogeneity(*P*/*I*^2^)	Begg’s test (*p*)
Blood loss			
DHS vs PFNA	2	0.00/98.3%	1.00
DHS vs GN	7	0.01/66%	0.76
DHS vs PCCP	3	0.00/82.9%	1.00
DHS vs FHR	3	0.04/69.4%	0.30
PFNA vs GN	1	NA	NA
PFNA vs PCCP	1	NA	NA
Operation time			
DHS vs PFNA	3	0.00/98.8%	0.30
DHS vs GN	11	0.00/91.5%	1.00
DHS vs PCCP	4	0.00/92.9%	0.73
DHS vs FHR	3	0.00/91.4%	1.00
PFNA vs GN	2	0.63/0%	1.00
PFNA vs PCCP	1	NA	NA

*P* < 0.05 indicates significant heterogeneity in Cochrane Q-test, publication bias in Begg’s test. *NA* not applicable

### Blood loss

**Fig. 3:**
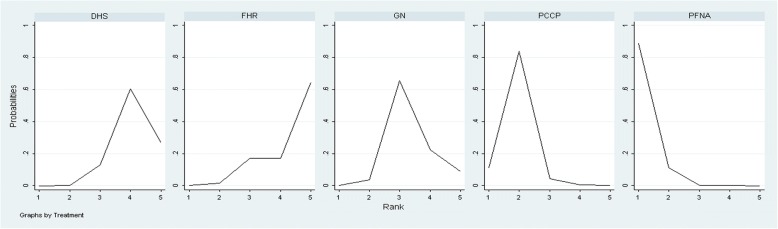
Ranking probability curves for blood loss during the operation. The graph displays the distribution of probabilities for each treatment. The *X*-axis represents the rank, and the *Y*-axis represents probabilities. The ranking indicates the probability that a particular treatment is the “best,” “second best,” etc

The results of our network meta-analysis comparing the five treatments regarding the outcome of blood loss are reported in Table 6. The outcome of five treatments ranked by the blood loss is also presented. Except for FHR, all interventions had greater effects on blood loss than DHS treatment. A relatively greater increase in blood loss was seen in DHS treatment than for PFNA (SMD 1.96; 95% CI, 1.014–1.963) or PCCP (SMD 1.26; 95% CI, 0.31–2.20) interventions (Table 6). This finding indicated that the PFNA intervention leads to the least blood loss among the five treatments studied. PFNA intervention consistently showed the highest probability of having the superior ranking position among all approaches, and PCCP use showed the second highest probability (Fig. 3).

**Table 6:** Results of the direct and indirect meta-analyses

a				
DHS	3.41(0.13, 6.70)	0.17(− 0.04, 0.38)	0.71(0.11, 1.31)	− 0.19(− 0.61, 0.22)
1.96(1.014, 1.963)	PFNA	− 0.43(− 0.81, − 0.04)	0.93(0.50, 1.37)	NA
0.26(− 0.35, 0.87)	− 1.70(− 2.74, − 0.66)	GN	NA	NA
1.26(0.31, 2.20)	− 0.70(− 1.87, 0.46)	1.00(− 0.10, 2.09)	PCCP	NA
− 0.23(− 1.26, 0.81)	− 2.19(− 3.60, − 0.77)	− 0.49(− 1.69, 0.71)	− 1.49(− 2.90, − 0.08)	FHR
b				
DHS	1.24(− 1.36, 3.84)	0.21(− 0.12, 0.54)	0.34(− 0.49, 1.16)	− 0.12(− 0.92, 0.67)
0.75(− 0.02, 0.75)	PFNA	− 0.30(− 0.60, 0.01)	0.94(0.50, 1.37)	NA
0.25(− 0.26, 0.77)	− 0.49(− 1.3, 0.33)	GN	NA	NA
0.61(− 0.20, 1.44)	− 0.14(− 1.16, 0.88)	0.35(− 0.59, 1.32)	PCCP	NA
− 0.12(− 1.15, 0.91)	− 0.86(− 2.15, 0.42)	− 0.37(− 1.52, 0.78)	− 0.72(− 2.03, 0.59)	FHR

a Comparison of blood loss between 5 treatments. b Comparison of operation time between 5 treatments. Results of direct comparisons are listed in the upper triangle, and the estimation was calculated as the row-defining treatment compared with the column-defining treatment. Results of network meta-analysis are listed in the lower triangle, and the estimation was calculated as the column-defining treatment compared with the row-defining treatment

### Operation time

**Fig. 4:**
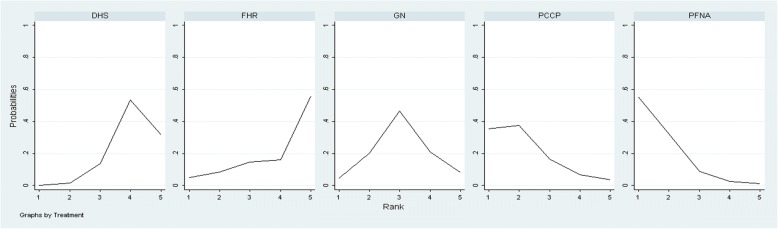
Ranking probability curves for operation time. The graph displays the distribution of probabilities for each treatment. The *X*-axis represents the rank, and the *Y*-axis represents probabilities. The ranking indicates the probability that a particular treatment is the “best,” “second best,” etc

Table 6 shows the results of the network meta-analysis on the outcome of operation time. Except for FHR, all interventions had greater effects on operation time than DHS treatment. A relatively longer operation time was observed for DHS than for PFNA (SMD 0.75; 95% CI, − 0.02–0.75) or PCCP (SMD 0.61; 95% CI, − 0.20–1.44) interventions. A significantly shorter duration was observed for the PFNA approach than for GN (SMD − 0.49; 95% CI, − 1.3–0.33), or FHR use (SMD − 0.86; 95% CI, − 2.15–0.42). Therefore, PFNA, PCCP, and GN interventions resulted in significantly greater decreases in operation time. Figure 4 shows that among the five treatments, PFNA treatment exhibited the highest probability of reducing the operation time, and PCCP treatment had the second highest probability.

## Discussion

This network meta-analysis reviewed existing evidence for the treatment of intertrochanteric hip fractures, with the aim to synthesize the evidence and to confirm decision-making in the absence of direct comparisons. Almost all previous RCTs or meta-analysis studies were evaluations of direct comparisons among DHS, GN, PFNA, and PCCP approaches. Few studies compared FHR with these treatments. Our findings integrate these previous comparisons.

With the rapid increase in the aging population, intertrochanteric hip fractures have shown an increasing trend. This type of fracture is a worldwide health problem, leading to serious medical consequences that interfere with quality of life and mortality [37]. During the past decades, this type of fracture has resulted in increased economic burden and has led to increased consumption of health care resources [38]. Nevertheless, considerable debate and controversy still exist regarding the optimal method and device for fixation of intertrochanteric fractures.

The DHS, which is an extramedullary fixation device, has previously been considered the gold standard treatment for intertrochanteric fractures [39]. However, this intervention, although technically simpler, requires a considerable amount of exposure and ambient soft tissue stripping, which can lead to heavy bleeding [40]. The disadvantages of traditional internal fixation, such as increased blood loss, postoperative pain, and slow function recovery, led to the emergence of a minimally invasive surgical option as an alternative treatment technique [41]. PCCP is a new type of minimally invasive surgical device. It consists of a biaxial, two parallel femoral neck screws, and three cortical screws. We can insert the screws through two 2-cm incisions percutaneously. Compared with DHS implants, it can reduce the damage of the lateral cortex and lateral femoral muscles. GN is an intramedullary implant with a short lever arm and a small bending moment. Compared with DHS implants, this implant has two advantages: The relatively semi-closed operation through a small incision does not require exposure of the fracture site; in addition, sliding screws can generate pressure on the proximal medial cortex to increase the stability of internal plants and fracture site. Therefore, inserting GN nails helps to reduce blood loss and shorten the operation time. PFNA intervention involves an innovative implant developed by the AO/ASIF group that is based on a proximal femoral nail (PFN) with a special helical blade. We found that PFNA treatment leads to the lowest amount of blood loss and the shortest operation time among the five treatments.

After PFNA, the amount of blood loss for each method ranked in the following increasing order: PCCP, GN, FHR, and DHS. Less blood loss during an operation leads to fewer incidences of allogenic blood and reduces potential risks of transfusion reactions, disease transmission and immunomodulation [42–44]. The risk of a serious or fatal transfusion-transmitted disease is approximately 3 in 10,000, while the chance of a minor allogenic reaction is 1 in 100 [42]. Regarding the reduction in operation time, PFNA treatment was also ranked as the top intervention. A shorter surgical time reduces the risk of minor or major anesthesia problems and blood loss, which helps to prevent the occurrence of anemia and hypoalbuminemia and improves patient recovery. Additionally, from a mechanical point of view, adequate evidence has demonstrated the superiority of the helical blade over a sliding hip screw for head stabilization and fragment sliding. The distinctive design consists of a large surface and an incremental core diameter, assuring maximum compaction and optimal hold in the bone. Because the PFNA blade can induce compaction of cortical bone, the increased rotational and angular stability can resist rotation and varus collapse of the head, as shown in cadaveric studies [45]. Therefore, PFNA, one type of modified minimally invasive implant technique used for internal fixation in elderly patients, is generally accepted in modern treatment of orthopedic trauma [46, 47].

The advantages of our analysis are that we simultaneously compared more than two treatments in the same analysis and provided relative effect estimates for all treatment comparisons, even for those that have not been directly compared before; thus, for each treatment, we estimated the probability that the given treatment is optimal. However, there are several limitations of our network meta-analyses that need to be acknowledged. First, we only analyzed total blood loss and surgical time because these were the most frequently reported outcomes in all included studies. However, many other outcomes should be considered, such as postoperative functional status, radiation time, complication rate, and fixation failure. Additional clinical trials and longitudinal studies with these outcomes may be reported in the future, enabling similar analyses. Second, statistical heterogeneity was moderate; however, most comparisons had wide 95% CIs and included values that indicated very high or no heterogeneity. Existing uncertainties regarding the potential heterogeneity of these studies should be explored.

## Conclusion

In conclusion, PFNA is biomechanically and biologically superior to other implant techniques for treating intertrochanteric hip fractures. It provides stable intramedullary fixation to resist varus collapse, leading to the lowest amount of blood loss and the shortest operation time.

## Abbreviations

CI: Confidence interval
CNKI: Chinese National Knowledge Infrastructure
DHS: Dynamic Hip Screw
FHR: Artificial Femoral Head Replacement
GN: Gamma Nail
NA: Not applicable
PCCP: Percutaneous Compression Plate
PFNA: Proximal Femoral Nail Anti-rotation
RCT: Randomized Controlled Trial
SMD: Standard mean difference
